# Acute liver failure due to concomitant arterial, portal and biliary injury during laparoscopic cholecystectomy: is transplantation a valid life-saving strategy? A case report

**DOI:** 10.1186/1754-9493-3-22

**Published:** 2009-09-15

**Authors:** Lucas McCormack, Emilio G Quiñonez, Pablo Capitanich, Sara Chao, Victor Serafini, Nicolas Goldaracena, Ricardo C Mastai

**Affiliations:** 1 The Hepato-Pancreato-Biliary and Liver Transplantation Unit, General Surgery Service, Hospital Aleman from Buenos Aires, Argentina

## Abstract

**Background:**

Combined iatrogenic vascular and biliary injury during cholecystectomy resulting in ischemic hepatic necrosis is a very rare cause of acute liver failure. We describe a patient who developed fulminant liver failure as a result of severe cholestasis and liver gangrene secondary to iatrogenic combine injury or the hepatic pedicle (i.e. hepatic artery, portal vein and bile duct) during laparoscopic cholecystectomy.

**Case presentation:**

A 40-years-old woman underwent laparoscopic cholecystectomy for acute cholecystitis. During laparoscopy, a severe bleeding at the liver hilum motivated the conversion to open surgery. Many sutures were placed across the parenchyma for bleeding control. After 48 hours, she rapidly deteriorated with encephalopathy, coagulopathy, persistent hypotension and progressive organ dysfunction including acute renal failure requiring hemodialysis and mechanical ventilation. An angiography documented an occlusion of right hepatic artery and right portal vein. In the clinical of acute liver failure secondary to liver gangrene, severe coagulopathy and progressive secondary multi-organ failure, the patient was included in the waiting list for liver transplantation. Two days later, the patient was successfully transplanted with initial adequate liver graft function. However, she developed bilateral pneumonia and severe gastrointestinal bleeding and finally died 24 days after transplantation due to bilateral necrotizing pneumonia.

**Conclusion:**

The occurrence of acute liver failure due to portal triad injury during laparoscopic cholecystectomy is a catastrophic complication. Probably, the indication of liver transplantation as a life-saving strategy in patients with late diagnosis, acute liver failure, severe coagulopathy and progressive secondary multi-organ failure could be considered but only minimizing immunosuppressive regimen to avoid postoperative infections.

## Introduction

Acute liver failure (ALF) is an uncommon condition in which the rapid deterioration of liver function results in coagulopathy and alteration in the mental status of a previously healthy individual [1]. Although some patients will improve spontaneously, some others will finally develop multisystem organ failure, which often is observed in the context of a hyperdynamic circulatory state that mimics sepsis. While a good spontaneous prognosis can be expected in some patients, in critically ill patients (i.e. APACHE II score >20) survival without liver transplantation is very unlikely [2]. Moreover, the risk of mortality increases with the development of any of the complications, which include cerebral edema, renal failure, adult respiratory distress syndrome, coagulopathy and infection.

Combined iatrogenic vascular and biliary injury during cholecystectomy (i.e. lesions of the bile duct with hepatic artery +/- portal vein) resulting in ischemic hepatic necrosis is a very rare cause of ALF [3-6]. Only few cases have been reported requiring emergency liver transplantation as a consequence of non-biliary complications (i.e. injury involving the hepatic artery +/- portal vein) during laparoscopy without optimistic results [6]. One patients presenting with a combined biliary and vascular injury recognized during surgery underwent total hepatectomy with portosystemic shunting and subsequent successful liver transplantation within the first 24 hours [5]. As only few case reports have been published, there are no systematic studies examining the best treatment strategy in patients with devastating intra-operative injury of the liver hilum [7]. To our knowledge, there is no report of unrecognized combined biliary and vascular injury during cholecystectomy treated with emergency liver transplantation as a life-saving procedure. We describe a patient who developed ALF as a result of severe cholestasis and right hemi-liver gangrene secondary to iatrogenic combine injury or the hepatic pedicle (i.e. hepatic artery, portal vein and bile duct) during laparoscopic cholecystectomy.

## Case presentation

A previously healthy 40-years-old woman underwent laparoscopic cholecystectomy for acute cholecystitis. Intraoperative cholangiography was not performed. After clipping gallbladder hilum structures and during removal of the gallbladder, a severe bleeding at the liver hilum motivated the conversion to open surgery. Many sutures were placed across the parenchyma at the site of the right portal vein entrance into the liver and in the gallbladder bed for bleeding control. Finally, the gallbladder was completely removed and biliary injury was undiagnosed. On the first postoperative day (POD) the patient developed elevation of liver function test and with presumption of right bile duct injury decision was made to transfer the patient to a tertiary center.

On intensive care unit admission (48 hours after onset of injury), she presented signs of shock (arterial pressure 80/60 mm Hg, heart rate 130/minute, anuria and dyspnea). Within hours, she rapidly deteriorated with encephalopathy, persistent hypotension needing noreprinefrin and progressive organ dysfunction including acute renal failure requiring hemodialysis and mechanical ventilation. Blood laboratory revealed elevated liver function test (AST: 12.167 IU/L; ALT: 3.838 IU/L and alkaline phosphatase: 354 IU/L), metabolic acidosis (serum lactate: 6 mmol/L) and a marked systemic inflammatory response syndrome (APACHE II score: 25 points). Impaired liver function was reflected by cholestasis (Total bilirubin: 20 mg/dL and direct bilirubin: 12 mg/dL) and reduction of coagulation activity parameters (prothrombin time: 18% and international normalized ratio: 4.5). An abdominal ultrasound revealed a heterogeneous right liver without dilatation of intra and extra-hepatic bile ducts. A computed tomography without contrast confirmed absence of intra-abdominal fluid collection. As a combined iatrogenic vascular and biliary injury of the right liver pedicle was suspected and with the intention to determine the precise extent of the vascular injury, an angiography was performed documenting an occlusion of right hepatic artery and right portal vein. Based on these findings and in the clinical context of a patient with liver gangrene, severe coagulopathy and progressive secondary multi-organ failure, she was immediately included in the emergency status of the waiting list for liver transplantation.

**Figure 1:**
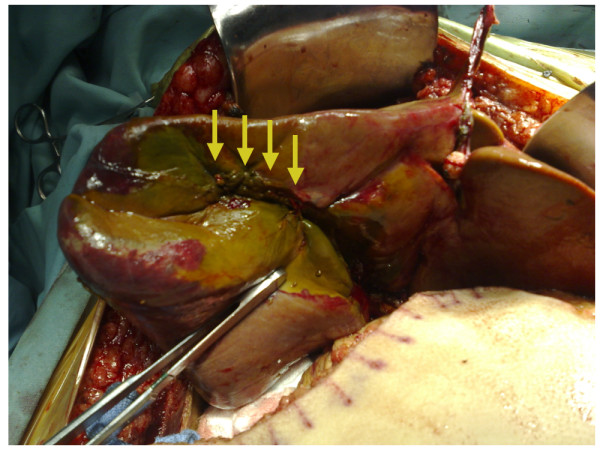
**Macroscopic aspect of the gangrenous right lobe in a liver with cholestasis at laparotomy**. Note multiple sutures placed in the gallbladder bed (arrows).

**Figure 2:**
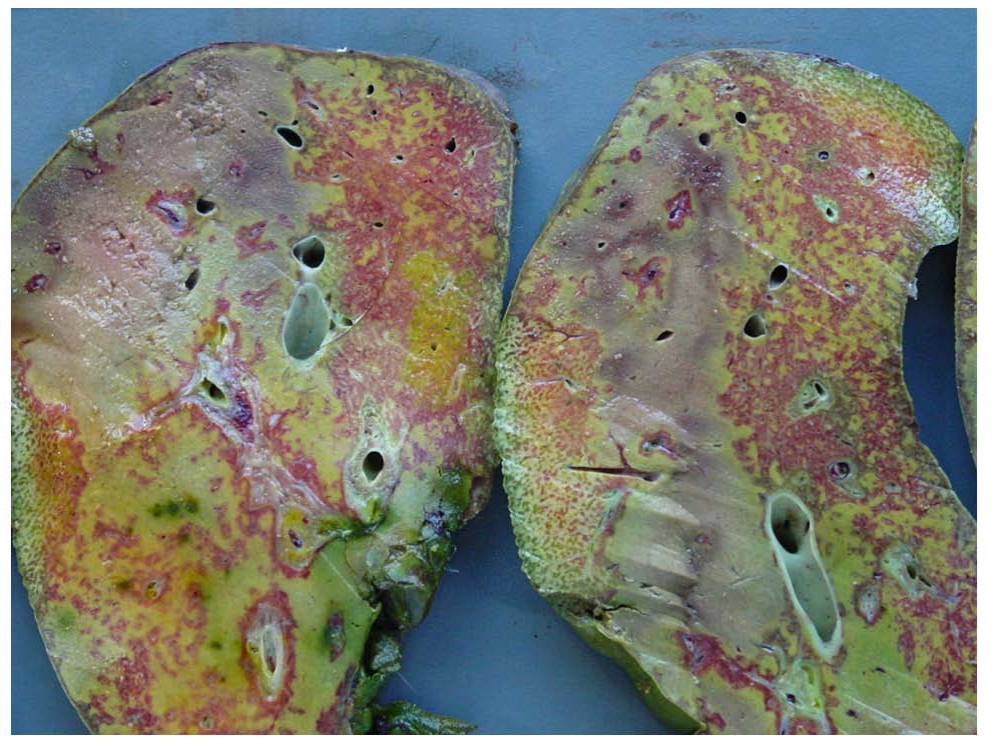
**Sections of the right liver lobe**. Note the parenchyma infarction with diffuse necrosis combined with hemorrhagic infiltration and cholestasis.

Four days after the onset of the liver injury, a cadaveric organ became available. At the time of transplantation, she was hemodinamically stable without signs of systemic infection that contraindicate liver transplantation. She had spontaneous epistaxis needing nasal packing and diffuses bleeding at each site of venous puncture. The preoperative laboratory blood test showed impairment of coagulation activity parameters (prothrombin time: <10% and international normalized ratio: 5.5) and transplantation was performed with continuous intra-operative hemodialysis. At laparotomy, a wound infection was detected. The macroscopic aspect of the right liver was gangrenous with the presence of severe cholestasis (Figure 1). Meticulous exploration of the pedicle demonstrated ligature and division of the right hepatic artery. The common bile duct was divided and resected up to the bifurcation. Necrotic liver parenchyma was identified in the area of the hemostatic sutures. The liver transplantation was performed using the standard technique with preservation of the native vena cava (Piggy-back technique) without intra-operative complications. The macroscopic aspect of the right liver parenchyma demonstrated infarction with hemorrhage and cholestasis (Figure 2). Basiliximab and corticoids were used for initial immunosuppression regimen. The patient recovered satisfactorily with good function of the liver graft and mechanical ventilation was removed on POD 4. But on POD 7 she developed bilateral pneumonia and severe gastrointestinal bleeding of unknown origin. Finally, she died on POD 24 due to bilateral necrotizing pneumonia secondary to multiresistent *Pseudomonas aeruginosa *infection with functioning liver graft.

## Discussion

Many lessons can be learnt to prevent the occurrence of this iatrogenic combined injury of the bile duct, hepatic artery and portal vein during laparoscopic surgery for a benign disease.

The delay in the correct diagnosis and the elapsed period between the time of a concomitant vascular and bile duct injury and the time of referral are factors that could negatively influence postoperative outcome. In this case, ligation and division of the bile duct was unrecognized neither during laparoscopy nor during converting laparotomy. An intra-operative cholangiography could eventually diagnose the bile duct division early during surgery preventing the consecutive injury in the vascular liver hilum. Although the utility of routine cholangiography to prevent biliary injuries during laparoscopic cholecystectomy is still under debate, most would agree to perform it during cholecystectomy in selective complicated cases needing conversion laparotomy or when multiple hemostatic sutures are placed at the liver hilum. A correct intraoperative diagnosis with a biliary decompression or a bilio-enteric reconstruction could avoid progressive cholestasis that contributed negatively to the occurrence of the liver failure despite the already established necrosis.

Unfortunately, the concomitant vascular and biliary injury was unrecognized and this patient was referred two days later presuming a single right bile duct injury. The sutures of the liver bed for bleeding control as the only intra-operative event in combination with the absence of dilatation of the intra-hepatic bile ducts in the left side in non-invasive imaging led to misdiagnose the complete hepatic duct occlusion. In addition, during initial work-up, the endoscopic cholangiography to document the biliary tree was precluded to avoid biliary sepsis, fact that could potentially contraindicate liver transplantation or eventually complicate postoperative outcome. Therefore, as only right pedicle injury at the liver hilum was suspected, the probability of a complete division of the common bile duct was not considered before transplantation. Our initial diagnosis was complete right hepatic pedicle occlusion and thus, we assume that the ALF was secondary to an acute ischemic reduction in the functional liver mass even in the presence of a well-vascularized left hemi-liver. In this scenario with coagulopathy and poor patient condition, a right hepatectomy was not considered as a good surgical option. Moreover, if the patient deteriorates after partial hepatectomy due to uncontrolled bleeding or postoperative infectious complication, the chance to be treated with delayed liver transplantation would be completely lost. The rapid progression to ALF and secondary multi-system organ failure motivated the immediate inclusion in the liver transplant waiting list. Another question could be what to do if complete transection of the common hepatic duct would be detected before transplantation: should we still go for liver transplantation or emergency right hepatectomy? Only one case has been reported treated with emergency right hepatectomy and Roux-en-Y left hepatojejunostomy in the acute setting after concomitant vascular and biliary injury, but this patient finally died of postoperative uncontrolled sepsis [7]. In contrast to our case, diagnosis was performed early after injury when inflammatory systemic response was not present and hepatic function was not impaired except for a rise in liver transaminases without coagulopathy.

A recent report propose that total hepatectomy with portosystemic shunting and subsequent cadaveric liver transplantation could be a good strategy for patients presenting with devastating portal transection recognized intra-operatively [5]. Perhaps, this total hepatectomy performed within the first hours could prevent the consequent multiorganic failure but this benefit is probably lost when diagnosis occurred late after surgery. Unfortunately, the literature lacks information regarding the better therapeutic strategy to be used in these complex injuries leading to ALF when diagnosis is performed too late and severe systemic inflammatory response is already established.

Unfortunately, liver transplantation is rarely successful after iatrogenic combined vascular and biliary injury of the liver hilum. When referral occurs too late, salvage liver transplantation usually is not feasible due to the presence of septic complications. In exceptional cases receiving a liver transplantation, infection is usually the final postoperative complication leading to patient death in patient with functioning liver grafts [6]. Therefore, based on published data, it is difficult to address the question whether liver transplantation is a reasonable option in patients with combined arterial, portal and biliary injury during laparoscopic cholecystectomy. Perhaps, the key factor to improve outcome could relay in early liver transplantation with very low immunosuppression to avoid infection in these patients.

Early referral of primary surgeon is probably the most important factor improving patient survival after this devastating biliary injury. The construction of adequate referral networks for the early treatment of severe surgical complications is paramount for patients requiring surgery in primary centers. This well-established strategy will provide a fast referral with early proper treatment to these critical patients. Unfortunately, a low donation rate in our country motivated that liver transplantation was performed two days after decision was made increasing the probability of nosocomial infection in a critically ill patient.

The real incidence of this complex injury is unknown. Probably, many cases have occurred but remain unrecognized for the surgical community due to the lack of referral or to a high mortality rate before the patient reach the tertiary center. As autopsy is performed exceptionally today in most centers, the underlying iatrogenic injury is probably not documented in most cases. Legal issues probably also contribute to an under-registry of this dramatic complication. Finally, the lack of reports in the literature of patients with successful outcome following liver transplantation can eventually lead to underestimation of emergency liver transplantation as a potential therapeutic tool.

Finally, the most important message relay on the role that surgical community and the public interest has on patient safety. National surgical societies and regional committees worldwide should develop and implement standards of excellence to ensure the highest quality of patient care through accreditation programs. They should encourage a minimum level of competence for surgical care of patients undergoing surgical procedures in primary centers. This political strategy will provide an external source for evaluating patient safety during surgical practice.

## Conclusion

The occurrence of acute liver failure due to portal triad injury during laparoscopic cholecystectomy is a catastrophic complication. Perhaps, the indication of liver transplantation as a life-saving strategy in a limited number of patients with late diagnosis, acute liver failure, severe coagulopathy and progressive secondary multi-organ failure could be considered but only minimizing immunosuppressive regimen to avoid postoperative infections.

## Consent

Consent was received from relatives (daughter) of the patient. A copy of the written consent is available for review by the Editor-in-Chief of this journal.

## Competing interests

The authors declare that they have no competing interests.

## Authors' contributions

LM: Design and drafted the manuscript EGQ: Helped to draft the manuscript. PC: Have made substantial contributions concerning acquisition of data. SC: participated in the design of the study. VS: Helped to draft the manuscript and acquisition of data. NG: participated in data collection, coordination and helped to draft the manuscript. RCM: Participated in the coordination and helped to draft the manuscript. All authors read and approved the final manuscript.
